# Nurturing futures through the maternal microbiome

**DOI:** 10.1111/1467-9566.13828

**Published:** 2024-08-07

**Authors:** Roberta Pala, Katherine Kenny

**Affiliations:** ^1^ Sydney Centre for Healthy Societies The University of Sydney Sydney New South Wales Australia

**Keywords:** care, gut health, microbiome, more‐than‐human, motherhood

## Abstract

Recently there has been growing recognition of the productive and protective features of our microbial kin and the crucial role of 'commensal' microbes in supporting and sustaining health. Current microbiological and pharmacological literature is increasingly highlighting the role of maternal gut microbiomes in the long‐term health of both mothers and children. Drawing on the information and advice directed towards Australian parents from conception through the first years of a child's life, we consider its messaging about the need to secure for the foetus/future‐child an enduring, optimal state of health by managing the maternal microbiome. We argue that this post‐Pasteurian trend gives rise to relations of care that are, at once, newly collective and more‐than‐human—but also disciplinary in ways that position the maternal microbiome as a new site of scrutiny that disproportionately responsibilises and burdens mothers. We notice how microbiome research is used both to reframe motherhood as a form of micro(bial)‐management and to maintain motherhood as a medicalised process. The feminist and more‐than‐human potential that this research can provide is missing in the way these resources are presented to parents.

## INTRODUCTION

Once the exclusive domain of biomedical scholarship, the microbiome has become an increasingly common object of scientific study, popular fascination and health intervention. Implicated in everything from periodontal disease, obesity, mood and mental health to inflammation, immunity and metabolism, the microbiome is now widely recognised as fundamentally constitutive of our embodied humanity as well as our current—and *future*—health. Made up of approximately 39 trillion microbes that live within and upon us, any one person’s microbiome contains bacterial, viral and fungal cells that outnumber human cells by a ratio of three to one. Increasingly, biomedical scholarship has highlighted various entanglements between one’s microbiome and their environment, diet, lifestyle and socioeconomic status (Gacesa et al., 2022). Importantly, the microbiome is also heavily dependent on one’s exposome, especially the conditions of gestation, birth, infancy and early childhood (Tamburini et al., 2016). As such, the long‐term health of foetuses, babies and children has been positioned as subject to maternal influence through ‘lifestyle’ decisions as they are mediated through the gut (EbioMedicine, 2021; Yao et al., 2020), reinscribing some of the normative pressures upon mothers across microbial and temporal scales (Camacho‐Morales et al., 2021; Hanson, 1998; Wolf, 2010; Yao et al., 2020).

In this paper, we look at the advice and information directed towards parents in New South Wales (NSW), Australia from conception through the first year of a child’s life and its messaging about the need to actively manage and maintain the child’s relations with ‘good’ microorganisms in order to secure the future, lifelong health and wellbeing of the child. We consider how this future orientation of caring for the microbiome establishes parenting (the act of culturing and nourishing future life) as a practice that involves the management of human relations with microorganisms.

Our findings show firstly that the microbiological research examining the microbial bond between mother and child is beginning to translate into new parental guidelines, albeit sometimes only in a subtle way. How this research is presented to parents has implications not only for how maternal bodies become uniquely scrutinised but also for how motherhood becomes experienced as a form of micro(bial)‐management. Secondly, despite the understanding brought about by recent microbiological literature that it quite literally ‘takes a (more‐than‐human) village’ to raise healthy children, we highlight the burden that this knowledge brings onto the individual parent, especially mothers. We examine a range of parenting practices, from gestation through the first years of life and how they are being rearticulated at the microbial scale. We argue that alongside the more‐than‐human potential of the microbial turn, cultivating the optimal microbiome for the future health of the child can exert intensified disciplinary pressure on mothers to bring about the best possible future. We situate this condition within a wealth of already existing scholarship that analyses the medicalisation of motherhood and the enrolment of biological knowledges and regimes in the responsibilisation of women. As a result, not only is the microbiome deployed as a new site of scrutiny of mothers, but it is also selectively deployed in such a way as to maintain motherhood a medicalised domain.

Finally, our research shows that these parental guidelines situate microbial responsibilities on the mother in an extended timeline and space, conflating the microbial care that happens in the pregnant and breastfeeding body with the care that happens in the domestic space in the first years of the child’s life. After being strongly pressured to breastfeed due to the immunological benefits that lactation can offer through the microbiome, for example, it is still the mother that is encouraged to monitor wet and dirty nappies’ frequency and colour or to follow specific guidelines in the introduction of solid foods to optimise the child’s gut health. As always, the biological becomes conflated with the social and gendered pressures on the mother become biologized. This can result in the marginalisation of those who cannot cultivate their child’s microbiome in these prescribed ways, while also undermining more equal forms of parenting.

## PREDICTION, PREVENTION AND THE MEDICALISATION OF MOTHERHOOD

For most of human history, practices surrounding gestation, birth and parenting (largely mothering), fell firmly within the cultural, not medical realm. Since the mid‐19th century, though, motherhood has been drawn increasingly into the realm of medical authority and biomedical scrutiny (Martin, 1989; Wolf, 2010). In line with broader trends within Western biomedicine, pregnancy and motherhood increasingly became physical conditions subjected to various forms of expert intervention, bestowing upon medical professionals the authority to prescribe and control how women *ought* to behave as mothers from pregnancy onwards. The medicalisation of motherhood from conception, through birth and into the early parenting years has been attributed, at least in part, to the pervasiveness of risk culture (Burton‐Jeangros, 2011; Grant et al., 2018; Hallgrimsdottir & Benner, 2014; Lee, 2008; Possamai‐Inesedy, 2006; Wolf, 2010). This has become all‐encompassing in contemporary experiences of motherhood—at least in wealthy industrialised contexts—where mothering is burdened by formal and informal risk evaluations and mitigation responsibilities (Giles, 2012; Knaak, 2010). This is evident from conception where pregnancies are now considered to be either low or high risk, but not without it (Wolf, 2010) and continues through successive parenting decisions about breastfeeding versus bottle‐feeding, timing and extent of vaccinations, introduction of food and potential allergens and so on.

Feminist scholars have long combated these societal pressures—including the biomedicalisation of women’s bodies—by emphasising and advocating for the embodied expertise and authority of the pregnant person, of which the foetus is considered as an interdependent part. New biological research around the microbiome similarly proposes a symbiotic set of microbial relations between mother and child via the role of commensal bacteria. And, while this new scientific literature could therefore be presented as emancipating mothers from the medicalisation of reproduction, we notice that it instead fits into these same medicalised and risk‐based narratives about motherhood and health.

## MOTHERING THE MICROBIAL

Parental responsibilities have involved some form of management and awareness of the impact of microorganisms on human health for some time. In the mid‐1800s, bacteriology and the advent of germ theory had a profound impact on individual and collective behaviours around disease and health, both in the form of public health policies and in the form of private and domestic hygiene practices. Tomes (1998) explains how germ theory became popular especially through the so called ‘domestic science movement’, which brought significance to the domestic tasks of housewives and mothers and their role in maintaining a healthy environment through hygienic vigilance and germ avoidance. Women and mothers started being trained in home economics, a discipline based on the belief that basic education in household bacteriology would make women ‘…better wives and mothers’. The way in which the microbiome is used now to rethink mothers’ roles as managers of the health of the household, is in this sense, following a similar pattern of maternal responsibilisation. But, differently from germ theory, the microbiome is moving the hygienic vigilance within the body itself. It is the trillions of microorganisms within and upon their own bodies that mothers now need to monitor and govern not only for the sake of their own current health status, for the future health of their offspring, too.

New microbiological and pharmacological research focused on the microbial relations between mothers and offspring fits into a broader microbial turn that has shifted the discussion away from ideas about human bodies as discrete, individual and autonomous towards greater recognition of their entanglement with and dependence on the trillions of other organisms that make up our bodies (Fishel, 2017; Ironstone, 2019; Paxson, 2014). While initial attention to the microorganisms that are implicated in human health was aimed at controlling or fighting ‘bad’ microbes (i.e., a Pasteurian microbiopolitics), as was also the case in the context of germ theory, new microbiological studies are increasingly recognising the vital contribution of ‘good’ microbes for our health. As Paxson (2014) explains, a post‐Pasteurian microbiopolitics gives greater recognition to our entanglement with the microbial world—good, bad and neutral microbes alike—and the need to harness good microorganisms as allies against bad ones, rather than indiscriminately exterminating them all, usually through the use of overly broad‐spectrum antibiotics. Paxson theorises a way to live with microorganisms that moves beyond the Pasteurian pathogenic imaginary and is symbiotic and cooperative, instead.

Microbiome research has had a profound impact on understandings of pregnancies, birth, nurturing practices and medical interventions on maternal bodies. This microbial turn is already challenging concepts of individual health, human and non‐human (Shildrick, 2022) and the neat distinction between mother and foetus, replacing any potential competition with a recognition of mutual dependence (Mansfield, 2017; Takeshita, 2022). Microbes and humans depend on each other for survival and development and in the case of human reproduction, the child depends on the mother and her microbiota (i.e., the range of microorganisms hosted by the maternal body) for its immediate survival, development and long‐term health.

## MANAGING THE FUTURE

Much of the medical attention during pregnancy and the early stages of motherhood is increasingly directed towards the symbiotic relationship between humans and microbes and mother and child. This attention is based on the understanding that the transmission of maternal microbes to the offspring has crucial implications for the immunological, physiological and neurobiological development of the child possibly all the way into adulthood (Al Nabhani & Eberl, 2020; Bodden et al., 2021; Brodin, 2022; EbioMedicine, 2021; Mutic et al., 2017). This transmission begins in utero (Walker et al., 2017) and continues during birth (Mutic et al., 2017), throughout lactation and other early parenting practices (Camacho‐Morales et al., 2021). The research on these wide‐reaching relations between mothers, offspring and microbes throughout time—though often in very early stages—is giving rise to powerful claims that much about the *future life of the child*, specifically their health and even resilience, might depend on these very early moments of their life and on the parents’ and especially mothers’ health behaviours (Dominguez‐Bello et al., 2019).

In this microbiological literature, the temporal implications of the maternal management of microbiota are all‐encompassing, not only in the future‐oriented register of the health of the baby, but in relation to the past decisions and behaviours of the entire maternal line:

Infants are naturally born with their skin and mouth covered by maternal inocula and have swallowed these microbes, supported by the observation of both DNA and live bacteria in the meconium. Thus, we inherit the primordial microbiota from our mothers, grandmothers and further on the matrilineal line, with microbial vertical transmission extending back to earlier ancestors.

(Dominguez‐Bello et al., 2019, 1109)

While this astonishing perspective on human development and its entanglement with the microbial world speaks of a complex connection, of which we seem to know very little still; in a risk culture this knowledge acquires a more ominous tone, focussing on the dangers that are inherited together with this microbial legacy. Different ‘perturbations’ of the maternal microbiota are shown to increase health risks for the mother and the child especially. C‐section birthing and early antibiotic exposure are often presented as increasing risk of obesity for the offspring; formula‐feeding and excessive bathing of the baby are also listed as perturbing the child’s microbiota (Dominguez‐Bello et al., 2019). Perineal injury during delivery, post‐partum sleep deprivation, diet and newborn skincare practices all appear to ‘synergistically influence’ the microbiome and by extension the risks posed to the health of the baby (Mutic et al., 2017). This burgeoning body of research presents the future health of the child as dependent on women’s bodily practices—even before they become mothers—by way of ‘foetal programming’ (Camacho‐Morales et al., 2021). Words such as ‘programming’ and ‘induction’ suggest an anticipatory approach to optimising children’s microbiomes and the attendant risks of suboptimal management.

## EMBODYING RESPONSIBILITY

This new microbiological literature, together with epigenetic studies, has been problematising established understandings of individuals’ responsibilities for health and disease. In fact, it often moves beyond individual health ‘behaviours’ to consider the broader environmental contexts as implicated in how these microbial changes get ‘under the skin’ (Lappé & Jeffries Hein, 2021, p. 459). In this way, urbanisation or pollution or the use of antibiotics are all seen to impact microbial diversity, thereby increasing various health risks (Dominguez‐Bello et al., 2019). Nevertheless, the main site of intervention remains the maternal body, not the social contexts in which it lives and reproduces. Similar conclusions have been drawn from research on epigenetic studies of the mother‐child relation, which show how the intergenerational effects of maternal stress and trauma (Lappé & Jeffries Hein, 2021) centres the potentially harmful behaviours of mothers not only during pregnancy but across generations (Pentecost & Meloni, 2020). STS scholars such as Sarah Richardson (2021, 2017), Ruth Müller and Martha Kenney (Kenney & Müller, 2018; Müller & Kenney, 2021), have explored and critiqued how much epigenetic discourse is concerned with the maternal body and the way in which the ‘mother’ becomes the figure representing and mediating the whole environment. Through this scientific scholarship, these authors argue, ‘remarkably stereotypical notions of maternal agency and responsibility often travel … without much scrutiny and are, in the process, reinforced and solidified rather than critically questioned’ (Kenney & Müller, 2018, p. 808) According to Chiapperino and Panese (2018), this gendered framing of the biological effects of parenting, ‘is rather intrinsic to the material and experimental configuration of these scientific settings’ (1237).

While new microbiological knowledge further connects mothers and their offspring with micro and macro conditions, the primary point of intervention proposed thus far remains the mother. The maternal microbiome becomes a categoriser of bodies and practices—bodies at risk, risky practices, good/bad mothering, good/bad future health. Studies of microbiota fit into a context of professionalised nurturing, of medicalised parenting, of individualised maternal responsibility—as this new science explains how best to take care of your child. Paradoxically, the focus on the incredible life‐giving role of mothers and their microbiome as best suited to take care of their babies, is turned against women if not carried out in the ‘right’ way, according to medical experts. The burden of these interspecies relations thus becomes personal, carried by women, devoid of wider social and political obligations and the target of personalised intervention. Thus, the microbiome, despite *appearing* to distribute responsibility for nurturing early life beyond the mother, is used to place further individual responsibility on mothers, turning motherhood into a personal project of micro(bial) management and marginalising those that cannot sustain the burden of optimising not only their babies but billions of bugs that contribute to their wellbeing.

We will see that this individualised approach to optimising the child’s microbiota extends beyond pregnancy and breastfeeding. One way that this new body of research is presented to the public is through parenting resources, such as those distributed to new parents in NSW. Intended to guide parents—especially mothers—over the first year of a baby’s life, these resources extend the mother‐foetus microbial connection to subsequent parental practices such as the monitoring of the child’s exposure to dirt or external microorganisms and different approaches to the introduction to solid foods. They seemingly extend the scrutiny over pregnancy and breastfeeding to later stages of the child’s life, in many ways microbially tethering the mother to the child in an ongoing way. In acknowledging the distributed causes of child wellbeing across a wide range of microbial kin, this approach implicitly undermines other forms of distribution—for example the distribution of caring labour beyond the mother.

## METHODS

This paper draws empirical data gathered from the resources provided or sponsored by the NSW Government for women who are either trying to get pregnant and/or are already pregnant as well as to parents who have recently had a child. We looked at government websites such as nsw.gov.au/family‐and‐relationships/having children and health.nsw.gov.au, information packs given to parents of every baby born in a NSW hospital as part of the state‐sponsored ‘Baby Bundle’ and other resources and websites sponsored by the NSW government, such as Mothersafe (counselling service for women and their healthcare providers concerned about exposures during pregnancy and breastfeeding), the Australian Breastfeeding Association (ABA), the Australian parenting websites raisingchildren.net.au, pregnancybirthbaby.org.au and eatforhealth.gov.au.

We mapped these websites and resources to identify how scientific research on microbial‐maternal relations is impressed upon new parents in NSW. We wanted to consider how this new knowledge is organised in the specific context of parenting guidelines and how it is mobilised to deliver particular ideas about parenting roles and duties, especially for mothers. Of the seemingly infinite universe of information and advice presented to new parents, therefore, we focused on State‐sponsored information that directly engaged with the microbial world and its impact on human health by way of parenting practices.

We took an interpretive inductive approach to data analysis, guided by Clarke’s (2003) poststructuralist interpretation of the grounded theory method that moves beyond a positivist lens on the social and instead considers the complexities of data in nonreductive ways, emphasising the relations between human, nonhuman and discursive elements, the different interpretations of the research situation and the need to account for the multiple positions that can be taken in the data and any controversy that can arise from them. Specifically, we situated our data in the broader context of the pressures and scrutiny that new parents, especially mothers, experience. We attended to how the nonhuman is embodied and represented in State‐sponsored resources for parents and how it produces specific discourses around ‘good’ parenting as attentive nurturing and culturing of the microbial world. Going back and forth between the data and our analysis (Charmaz, 2017), we reflected on who is left out, what forms of parenting and nurturing are marginalised when doing parenting ‘right’ becomes a matter of microbial management.

## FINDINGS

When you are expecting a child in NSW, there is an almost overwhelming amount of information and array of services that the state government provides to mothers and parents in general. For example, *The First* 2000 Days *Framework* (NSW Health, 2019) policy directive outlines the strategies that the NSW health system needs to implement in order ‘to ensure that all children have the best possible start in life’ (1). The framework and the health initiatives it supports are based on new evidence showing that ‘the in utero experience of a baby followed by a child’s early life experience predicts their chances of succeeding at school, of doing well in life and of having chronic diseases as an adult’(8). The rapidly advancing literature on early development that this policy relies on, considers biological processes, global factors, social determinants of health such as poverty and inequality, family and community characteristics and individual factors (Moore et al., 2017). Importantly, it draws attention to areas that were not previously appreciated for having a role in development, such as the microbiome. In this report, the diverse ecology of microbes that make up the microbiome and the microbial axis that connects gut and brain are described as crucial for our physical and mental health including their beneficial functions in digestion, metabolism, hormone regulation, detoxification, immunity and fighting off ‘dangerous pathogens’ (Moore et al., 2017, p. 16).

Health initiatives that focus on the first 1000 or 2000 days of a child’s life emphasise the importance of monitoring and intervening in the microbial colonisation of the gut that happens between birth and the next two years, because it is in this small window that the gut microbiome is shaped for life (19). The factors that contribute to microbial colonisation—gestational age, antibiotic exposure, delivery mode, breastfeeding, formula milks, timing and types of solid foods—are stages and sites of interventions in the NSW health policy directive. The strategies mostly focus on parental (i.e., maternal) education, by extending ‘antenatal and birthing care [and] promoting access to evidence‐based parenting programs from pregnancy onwards’ (NSW Health, 2019, p. 22).

As mentioned earlier, the medicalisation of all aspects and stages of parenting has constructed motherhood as an intense site of monitoring. As this policy document shows, research on the first thousand days of child development including microbiological evidence on the maternal microbiome is being mobilised as a new site of health management over women and their bodies and as a new reason to promote even further scrutiny and surveillance.

### Eating (for) the future: Anticipatory management and bodily conduct

The NSW government’s guidelines and information packages on having a baby recommend taking care of one’s lifestyle and diet even before getting pregnant and most definitely throughout pregnancy. This is not new; risk anticipation has been a technique of scrutiny over mothers for some time and it has heavily impacted women’s choices around food during pregnancy and lactation (Burton‐Jeangros, 2011). What is new is the way in which microbiological studies are now recruited to further enforce prescribed behaviours, not only during pregnancy but even before conception, emphasising the long‐term implications that current choices in the maternal diet can have for the child’s future health. For example, in introducing pregnant women to the ‘pregnancy weight gain calculator’ tool on their website, the NSW government guideline reads: ‘what you put into your body before, during and after pregnancy can affect your baby’s health and development’ (NSW Government, 2023).

It is normal to gain weight during pregnancy… However, gaining too much weight can put you at risk of gestational diabetes and put your baby at greater risk of becoming overweight or developing metabolic syndrome later in life.

(Eat for Health)

Throughout pregnancy, women are urged to monitor their weight, eat a healthy diet, avoid specific foods and medications when possible to support their immunity and gut health, with direct implications that in doing so, they are also optimising the future of their foetus. The microbiome here is recruited not only to justify scrutiny over women’s lifestyle and diet but to enhance the bond between the mother and the foetus’s health. This microbial focus has added specificity and weight to the burden that is already put on mothers’ diet choices:

Evidence indicates that a healthy diet, including dietary supplements, can reduce birth defects and cognitive impairments, while an unhealthy diet promotes neuroinflammation as well as impaired neurotransmission and cognitive abilities, *potentially mediated* via alterations in the gut microbiome [emphasis added].

(Bodden et al., 2021)

In this microbiological research, this link between what mothers eat and their children’s future cognitive capacities isn’t necessarily proven—but as we see in this quote only *potentially mediated* by the microbiome.

The ‘Pregnancy care guidelines’ government document (Department of Health, 2020) enters into the specifics of the recommendations that need to be made to pregnant women in regard to their diet. Not only are women urged to avoid specific food (raw or undercooked meat, raw eggs, unpasteurised dairy, etc), but they are encouraged to follow specific dietary patterns, supported by evidence showing for example, that ‘A lower risk of childhood leukaemia is associated with maternal consumption of fruit …, vegetables and legumes’ (76) or that ‘higher maternal intake of all dairy products is associated with a reduced risk of eczema in babies… Maternal milk intake is associated with reduced risk of neural tube defects, asthma, allergic rhinitis and cow’s milk allergy in children’ (76). Diet recommendations also refer to vitamins and supplements, which are recommended from before conception (Pregnancy Birth and Baby, 2023c).

The recommendation to control ‘what you put into your body’ does not refer only to food, but also to medications, vaccines and the exposure to drugs and infections. The NSW government recommends contacting Mothersafe, a free telephone service for women in NSW, to gain more information on exposure risks during pregnancy and breastfeeding. The information is provided as a tool to serve the mother’s attempt to plan, manage and control their own health and the future health of the child. ‘When starting a family it’s important to optimise your health—to give your baby the best start in life’ (Royal Hospital for Women's PlaN Clinic).

While the wide reach of what could influence the health of the baby should suggest and this is explained in *The First* 2000 Days *Framework* mentioned earlier, a distribution of health determinants beyond the mother’s capacity—something more in the sphere of intervention of social and environmental policies for example—the easier and most immediate intervention seems to be one on the woman, her education and her body, a site already historically accustomed to medical scrutiny and moralisation. This continues with regards to delivery, which is possibly one of the experiences where women most heavily endure the pressures, expectations and recriminations associated with understandings of what is ‘best’ for the child.

### Moralising with microbes in planning for birth

Pregnant women in NSW are encouraged to devise their own birth plan when they are in the later stages of their pregnancy (Pregnancy Birth and Baby, 2023b). This is explained as a way to inform their doctor of the type of care they would like to receive and as a device that allows them to remain involved and feel more in control of the labour and birth. Most importantly, when thinking about the birth of their baby, mothers are asked if they would like a vaginal birth or an elective caesarean section.

The information offered by the NSW government on this issue seems to not explicitly recommend vaginal birth over caesarean. This is interesting for our discussion on the impact of microbiological research over reproductive choices. Recent studies on the neonatal gut microbiome in fact, argue that microbial colonisation begins at birth and that it differs dramatically based on the mode of delivery. This research also states that the healthy development of the baby is linked to its exposure to maternal vaginal, skin and milk microbiota (Wong et al., 2022). So vaginal delivery is the method that ensures this microbial transfer and exposure. Being born with caesarean section, instead, due to this potential microbial ‘defect’ (Khoruts, 2016) is reported to be associated with ‘higher risk of developing increasingly common maladies, such as inflammatory bowel disease, coeliac disease, atopy and autism, as well as obesity during the teenage years’ (Khoruts, 2016). The microbiological argument is that a C‐section breaks the microbial bond between mother and child and this seems to align with a moral judgement about the fitness of the mother to be a good parent, when the microbial bond is conflated with a parental bond of intimacy and care.

It is interesting to notice when microbiological evidence is deployed to support specific parental practices and when it is not and consider why that might be. As mentioned earlier, vaginal birth is not directly encouraged. On the contrary, c‐section rates have steadily increased worldwide and in NSW the proportion of women who gave birth for the first time via c‐section has increased from 31% in 2011 to 37.6% in 2021 (Australian Institute of Health and Welfare, 2023). Despite that, c‐section are still listed as potential risks in the State‐sponsored resources we analysed. The guidelines for pregnancy care that were cited earlier explicitly invite pregnancy‐care providers to help reduce the number of c‐sections. This also aligns with the new support of medical practitioners that antenatal classes that promote vaginal birth over c‐section, such as Calmbirth, have been gaining recently. Calmbirth classes are supposed to teach how to be in control of the birth process. They promote natural birthing methods over medical interventions. This framing of nature versus medicine sits in a broader discussion and feminist struggle around the medicalisation of birth. Feminist scholars such as Emily Martin have in fact argued that the medicalisation of birth—the increased number of c‐sections and the use of vacuum and forceps to assist delivery—has been a process that has progressively bypassed women’s role, agency and choice in the labour of birth (Martin, 1989). Calmbirth claims that its programme can have protective effects on post‐natal adjustment and potentially post‐partum depression, by allowing women to retain more control over the birth.

While this emphasis on vaginal birth aligns with the microbiological research previously cited, a recent new practice that promises to transfer the microbial benefits of vaginal births onto c‐section births, called vaginal seeding, remains debated and still at the margins of medical orthodoxy. Vaginal seeding refers to the practice of transferring vaginal‐type bacteria to babies delivered via c‐section using a gauze that has been pre‐incubated in the maternal vagina.

While women are told that it is their preference whether to have a vaginal delivery or a c‐section, the choice is not presented in neutral terms. It sits instead in a context of pervasive and subtle pressure on mothers to deliver their babies vaginally or to guilt‐trip those who end up delivering via c‐section for a reason or another. The increased rates of c‐section do not seem to be related to a de‐stigmatisation of this procedure. Interestingly, a practice such as vaginal seeding, which carries so much feminist potential to lighten the burden of the judgements attached to these decisions and can be framed as harm reduction in microbiological terms, remains controversial among medical professionals.

This shows that microbiome research is not taken up by these guidelines simply as new scientific insight into the relation between humans and microorganisms, but also and especially to reinforce the framing of birth as a medical practice to be carried out by medical professionals. This is also clear in the advice around the option of choosing a homebirth. While homebirth care is mostly provided by private midwives, there have been efforts in NSW to provide publicly funded homebirth options, on the basis that ‘there is clear evidence that a calm, homelike environment supports a woman’s sense of control and increases her comfort during labour and birth’ (NSW Government, 2020, p. 1). Despite this, the first key fact that a parent considering home birth is given in Australia’s leading pregnancy and baby website, is that: ‘It’s safest to have your baby in hospital’ (Pregnancy Birth and Baby, 2023a). We know that according to microbiological research the postnatal environment has important implications for the early colonisation of gut microbiota, therefore for the future health of the child. But in this case, a risk‐based medicalisation of birth takes precedence over the potentially emancipatory and microbiological implications of homebirths.

### Nourishment and normativity: Feeding practices in the first year of life

While the pressure to deliver vaginally is pervasive and moralised but not explicitly reinforced by the NSW government resources provided to women, not the same can be said for the choice between breastfeeding and formula‐feeding.

In NSW, women are strongly encouraged to breastfeed their babies. A NSW Policy Directive clearly requires NSW health organisations to promote, protect and support breastfeeding (NSW Health, 2018). The pressure on mothers to breastfeed comes from many directions. Women are linked to the services provided by the ABA, lactation consultants are available at the hospital following delivery and nurses at the local Child and Family Health Services will continue to encourage women to breastfeed in the following months. Personalised breastfeeding support is also available to those who have the time and the information on how to access it. According to the ABA:

Breastmilk contains about 200 different types of oligosaccharides (prebiotics), which provide food for the good bacteria in the gut. Our genes carry ‘memories’ from past generations … So, who we are today is a result of who our past generations were—up to three generations back at least! The breastmilk you provide for your baby has a positive impact on not only them but also their children!

(Australian Breastfeeding Association, 2022b)

The recommendation to breastfeed is explicitly related to the microbiological literature arguing that the good gut bacteria transferred through breastfeeding will have long‐lasting health outcomes for the child and potentially for the mother as well. This information is accompanied by the list of the health risks for mothers who don’t breastfeed and for children who are not breastfed. The ABA claims that not breastfeeding increases the risks of breast and ovarian cancer, type 2 diabetes and high blood pressure and heart disease and stroke for mothers. A baby who is not breastfed or is breastfed for a short period of time risks ‘gastrointestinal infections, respiratory infections, ear infections, SIDS, necrotising enterocolitis in premature babies, sepsis in premature babies, leukaemia, dental malocclusions, overweight and obesity, lower IQ’ (Australian Breastfeeding Association, 2023).

From a feminist perspective, breastfeeding as a social practice can be a contentious field. On the one hand, if we consider the fact that the medicalisation process of birth and motherhood began with the professionalisation of paediatricians as experts in overseeing feeding and pushing for artificial feeding (Wolf, 2010), the focus on breastfeeding can reflect a return of the expertise to the mother, a new sense of agency and control over motherhood to the mother. On the other hand, the microbiological literature supporting breastfeeding leaves the practice firmly within a medicalised and risk‐based embrace (Avishai, 2007). This research on the microbiome challenges the binary thinking that separated mother from foetus, which has contributed to normalising technological interventions such as c‐sections or formula feeding (Takeshita, 2022). But, as we are seeing, the reinstated mother‐foetus bond needs to be managed according to specific medical directives and is therefore valued only conditionally. Mothers are not only pushed to breastfeed but to *exclusively* breastfeed for the first 6 months and to follow the rest of the guidelines. While maternal milk is ‘nature at its best’, lactation remains a heavily scrutinised practice and microbiological knowledge is deployed to normalise this scrutiny. Mothers are left with only another extremely moralised alternative, formula feeding, at the cost of being shamed for it (Carlin, 2023).

Apart from breastfeeding, in the months following birth, there are other recommendations given to parents regarding the monitoring of the child’s gut microbiome. Microbiological research is therefore deployed to guide and assess parental practices beyond the maternal body. Its intervention moves from the internal environment of the maternal body to the domestic environment. The mother‐foetus bond gets extended across the domain of input (via breastfeeding) to the realm of urinary and faecal output. Here too, information resources are seemingly directed towards the same subject, that is, the mother.

Part of the education of a new parent is learning what is normal or not normal to find in a baby’s nappy. Parents are encouraged to keep track of the wet and dirty nappies, including both their frequency and colour. ‘This tells you a lot about your baby’s health’, says the Australian parenting website Raising Children (Poos and Wees, n.d.). Poo colours’ charts are often provided to new parents (Pregnancy Birth and Baby, 2022). Parents are told that problems relating to a ‘different’ looking poo can be due to a range of factors: oversupply of milk, lactose overload, food intolerance or allergy, jaundice, chronic diarrhoea, constipation, bacterial infections (Australian Breastfeeding Association, 2022a). Being familiar with the microbial ecosystem that influences your child’s health means moving in and out of boundaries between individual bodies and environments, between inside and outside, between cleanliness and dirt. Health becomes a multispecies affair, nappies new sites of microbial monitoring and the monitoring a mother’s role. This last point is not explicitly made, the poo guides are not directed to mothers, but they address a more general ‘you’ and ‘your’ baby. Here and there though, as nappy checks look different for breast‐fed babies and formula‐fed babies, suggestions like ‘try feeding from just one breast at a time until the breast is drained’ reveal the assumption that the target reader is in fact the mother. The same happens in reference to information on how to introduce babies to new foods when they are 6 months old. In fact, the attention to an infant’s gut and its relation to good and bad health, shifts to their diet once they start eating their first foods. The generic ‘you’ is given specific directions. Solid food should not be introduced too early because it can cause a reduction in breastmilk production and it can expose babies to more germs. It should be iron‐rich and nutrient dense food, ‘this is a wonderful opportunity to check your own diet and to ensure the whole family is eating a very healthy diet’ (Tresillian).

Despite the fears of allergic reactions, parents are encouraged to introduce allergens from 6 months of age and definitely before the first year of age. Allergens should be introduced one at a time to better monitor the child in case of adverse reactions. If there are no adverse reactions, then that same allergen needs to be presented to maintain exposure. As we mentioned earlier, increased allergy risks are reported to be associated with maternal diet before and during pregnancy. To lower the risk of allergies mothers are encouraged to keep breastfeeding, ‘although evidence for this is low’ (Australasian Society of Clinical Immunology and Allergy, 2020).

As they follow microbial paths and movements, the NSW sponsored resources that explain the development of a child’s immune system and gut health uncomplicatedly move from the biological role of maternal bodies to the social and parental role of mothers, presenting it with the same neutrality and necessity—eclipsing the normativity that underlies these directives.

## DISCUSSION: CULTIVATING THE MICROBIAL

There is a long tradition of research in the field of sociology of health, risk and family that explores the many ways in which motherhood is a heavily scrutinised, medically surveilled and socially moralised condition (Avishai, 2007; Burton‐Jeangros, 2011; Giles, 2012; Hallgrimsdottir and Benner, 2014; Hallgrimsdottir et al., 2017; Knaak, 2010; Lee, 2008; Pentecost & Meloni, 2018). This literature exposes the policing of parenting practices from pregnancy to feeding and providing ‘healthy’ food (Ballif, 2023), to the management of children’s free time (Clark and Dumas, 2020) and so on. It calls for resistance against the pressures and expectations of intensive motherhood, a condition characterised by a logic of ‘unselfish nurturing’ (Hays, 1996) and ‘imbued with the meanings of risk, danger, responsibility and constant reflexivity upon how well one cares for one's children’ (Lupton, 2011, p. 638). In this context, microbiome science could be seen for its hopeful potential to distribute this responsibilisation beyond the mother and beyond the human onto the trillions of microbial kin that populate our bodies and worlds. But this feminist potential remains illusory. Research on the microbiological relations between mothers and offspring would seem to bring back that active role to the mother in the medical realm, integrating her in the health outcomes of her baby. Guidelines and recommendations by the NSW health department push for the normalisation first and then optimisation of the microbiological bond between mothers and offspring as appropriate and desirable parental practice. Due to the ‘immune programming’ capacity that the maternal microbiome can have on the babies, mothers are asked to monitor and control their own diet even before becoming pregnant, negotiate between their needs and those of their babies in relation to the medications they take, preferably give birth vaginally but under specific limitations of time and place, breastfeed at least for the first 6 months, monitor the colour of their wet and dirty nappies, allow their babies to get dirty and put things in their mouths but also protect them from germs, bad microorganisms, vaccinate them on time and expose their immune systems to all allergens early.

Microbiological research trickles down to the everyday experiences of new parents. The management of the child’s relation to microorganisms is incorporated with the rest of maternal duties. We have seen this before with regards to other forms of biological regimes—that is, germ theory (Hallgrimsdottir & Benner, 2014; Tomes, 1998), epigenetics (Chiapperino & Panese, 2018; Lappé & Jeffries Hein, 2021; Pentecost & Meloni, 2018) and neuroscience (Macvarish et al., 2014). In all these instances, different forms of scientific findings were used to intervene in and police parenting practices and childcare, with the usual emphasis given to maternal behaviour. This research has been made to fit into a neoliberal understanding of motherhood as a totalising practice of risk management and avoidance (Wolf, 2010), of health optimisation both in the immediate and in the future tense. Uncertain microbial futures are expected to be controlled, protected or avoided by mothers.

The new research on the role of the microbiome in pregnancy and parenthood, within a risk culture and medicalisation, expands the sphere of responsibility beyond the human to the microbial, but also expands the sphere of medical and moral scrutiny over mothers to the micro and nano scale. The microbiome, despite appearing to distribute responsibility for nurturing early life beyond the mother, is used to place further individual responsibility on mothers, turning motherhood into a personal project of micro(bial) management and marginalising those that cannot sustain the burden of optimising not only their babies but billions of bugs that contribute to their wellbeing. With the authority and neutrality recognised to the ‘scientific’, the microbial in relation to mothers and children is deployed to increase the scrutiny to a nano scale. At the same time, the implication on which this research is based—the existence of trillions of non‐human bodies that influence human health—remains unexamined in its political potential. Paxson (2014) defines microbiopolitics as ‘the creation of categories of microscopic biological agents; the anthropocentric evaluation of such agents; and the elaboration of appropriate human behaviours vis‐a‐vis microorganisms engaged in infection, inoculation and digestion’ (17). How microbiome research is conducted shapes the conclusions it reaches. Microbiome research and its social implications are co‐constructed. How these conclusions are then presented to expecting women and new parents has implications not only for how maternal bodies become uniquely scrutinised but also for how motherhood is experienced as a micro‐management and site of new pressures and anxieties. By entering this realm, the microbiome is inevitably a political site.

Discourses that guide and monitor how to best parent have material implications; they are embodied. When these discourses present parenting as a form of nurturing futures through the microbial in neoliberal terms, as practices that individual parents, especially mothers, need to become experts about—other politics of cultivating microbial futures remain excluded. Little is said about the impact that these new microbiopolitical pressures exercise on those who can’t afford them—those who can’t access this information, those who can’t breastfeed, those who can’t stop taking some medications even while pregnant or breastfeeding, those who can’t be that discerning in their diet choices and then in that of their children. They are not only being ‘bad’ mothers but also potentially setting up their children to have bad health in their future.

Gender studies scholar Takeshita (2022) considers the microbiological research for its feminist potential to propose a symbiotic and ecological conceptualisation of pregnancy and reproduction. The author hopes that presenting reproduction as a bacterial event will allow to ‘displace the sexed physiology of reproduction and … move pregnant bodies into a more fluid space that opens the door for alternative approaches to scientific and feminist studies’ (Takeshita, 2022, p. 20). Takeshita is aware that using these scientific developments to rethink motherhood and the bond between mother and child can be problematic and could lead to ‘cooptation by antifeminist discourses’ (Takeshita, 2022, pp. 19–20). This literature could in fact appear to naturalise motherhood once again, when to emphasise the interdependence of bacterial‐maternal‐foetal bodies, an extra pressure is being put on the pregnant body to deliver the infant vaginally, have plenty of skin‐to‐skin contact and breastfeed to optimise and support this relation. Takeshita argues that as a holobiont, the motherfetus is political because it focuses on the entanglement between these human and microbial bodies and not their distinction and because it distributes agency over reproduction beyond the human. But what appears from the parental guidelines we analysed, is that human‐microbial relations have already been integrated within a risk culture that not only further burdens women with new and moralised responsibilities, but also further entrenches them within the domestic realm. In this sense then, the politics of this microbial care remain disappointingly all but progressive or forward‐looking.

The microbiological research on the maternal microbiome would suggest that nurturing is a more‐than‐human event, it extends beyond the individual to the microbial, beyond and between the internal world of the maternal body to the external environment. It gives renewed strength to the claim that it takes a village (human and more‐than‐human) to raise a healthy child. This could be an encouraging thought, for pregnancy and motherhood can be isolating and solitary experiences. But a more‐than‐human perspective is not necessarily progressive.

While gut health is presented as the product of external conditions (diet, lifestyle, exposure to medications, etc.), the responsibility over its management remains constrained within the maternal body and the domestic space. The microbial bond between mothers and babies is only stretched out to include parental and domestic practices that need not be unique responsibilities of mothers but carers in general, in this way undermining more equal forms of parenting and adding to the many unrealistic pressures already put on mothers.

In *The Microbial State*, Fishel (2017) invites us to think of microbes as objects of politics and suggests shifting the attention from individuals to interactions. The omnipresence of microbes, their fluid movements between bodies and borders, their essential even though still not fully understood influence on human and ecological health, makes their management (responsibility of mothers) more insidious and an endless obligation. Given the way in which the microbiome is being implicated in every aspect of human health, the imperative to mother the microbial risks tasking parents (mothers) with endless responsibility for microbial optimization.

Alternative and more equal ways to nurture and care would be possible if these microbial maternal relations were used to shift the attention from the mother’s individual body to the ecological and cooperative implications of human‐microbial relations, to distribute parental agency and responsibilities more broadly beyond the mother and beyond the human. But this perspective is missing in the resources presenting microbiological literature to mothers in NSW. The multispecies perspective that the microbiome evokes is not inherently good nor emancipatory. In this case it has further entrenched maternal biopolitics. A political sensitivity that asks who is left behind and who carries the heaviest burden is needed to shed a light over the dark side of the microbial and the multispecies.

## AUTHOR CONTRIBUTIONS

**Roberta Pala**: Conceptualization (equal); writing – original draft preparation (lead); writing – review and editing (lead). **Katherine Kenny**: Conceptualization (equal); writing – original draft preparation (supporting); writing – review and editing (supporting); funding acquisition (lead); project administration (lead).

## Data Availability

Data available on request due to privacy/ethical restrictions.
